# Perioperative water and electrolyte balance and water homeostasis regulation in children with acute surgery

**DOI:** 10.1038/s41390-023-02509-1

**Published:** 2023-02-09

**Authors:** Daniel N. Roberts, Paula Vallén, Maria Cronhjort, Tobias Alfvén, Gabriel Sandblom, Susanna Tönroth-Horsefield, Boye L. Jensen, Per-Arne Lönnqvist, Robert Frithiof, Mattias Carlström, Rafael T. Krmar

**Affiliations:** 1 Sachsska Children and Youth Hospital, Stockholm, Sweden; 2 Department of Anesthesia and Intensive Care, Södersjukhuset, Stockholm, Sweden; 3 Department of Clinical Science and Education, Södersjukhuset, Karolinska Institutet, Stockholm, Sweden; 4 Department of Global Public Health, Karolinska Institutet, Stockholm, Sweden; 5 Department of Surgery, Södersjukhuset, Stockholm, Sweden; 6 Department of Biochemistry and Structural Biology, Lund University, Lund, Sweden; 7 Department of Cardiovascular and Renal Research, Institute of Molecular Medicine, University of Southern Denmark, Odense, Denmark; 8 Department of Urology, Odense University Hospital, Odense, Denmark; 9 Department of Pediatric Perioperative Medicine and Intensive Care, Astrid Lindgren Children’s Hospital, Karolinska University Hospital, Stockholm, Sweden; 10 Department of Physiology and Pharmacology, Karolinska Institutet, Biomedicum 5B, Stockholm, Sweden; 11 Department of Surgical Sciences, Anesthesiology, and Intensive Care, Uppsala University, Uppsala, Sweden

## Abstract

**Background:**

Hospital-acquired hyponatremia remains a feared event in patients receiving hypotonic fluid therapy. Our objectives were to assess post-operative plasma-sodium concentration and to provide a physiological explanation for plasma-sodium levels over time in children with acute appendicitis.

**Methods:**

Thirteen normonatremic (plasma-sodium ≥135 mmol/L) children (8 males), median age 12.3 (IQR 11.5–13.5) years participated in this prospective observational study (ACTRN12621000587808). Urine was collected and analyzed. Blood tests, including renin, aldosterone, arginine-vasopressin, and circulating nitric oxide substrates were determined on admission, at induction of anesthesia, and at the end of surgery.

**Results:**

On admission, participants were assumed to be mildly dehydrated and were prescribed 50 mL/kg of Ringer’s acetate intravenously followed by half-isotonic saline as maintenance fluid therapy. Blood tests, urinary indices, plasma levels of aldosterone, arginine-vasopressin, and net water-electrolyte balance indicated that participants were dehydrated on admission. Although nearly 50% of participants still had arginine-vasopressin levels that would have been expected to produce maximum antidiuresis at the end of surgery, electrolyte-free water clearance indicated that almost all participants were able to excrete net free water. No participant became hyponatremic.

**Conclusions:**

The use of moderately hypotonic fluid therapy after correction of extracellular fluid deficit is not necessarily associated with post-operative hyponatremia.

**Impact:**

- Our observations show that in acutely ill normonatremic children not only the composition but also the amount of volume infused influence on the risk of hyponatremia.
- Our observations also suggest that perioperative administration of hypotonic fluid therapy is followed by a tendency towards hyponatremia if extracellular fluid depletion is left untreated.
- After correcting extracellular deficit almost all patients were able to excrete net free water. This occurred despite nearly 50% of the cohort having high circulating plasma levels of arginine-vasopressin at the end of surgery, suggesting a phenomenon of renal escape from arginine-vasopressin-induced antidiuresis.

## Introduction

Hyponatremia, defined as plasma-sodium (*P*_Na_) concentration <135 mmol/L, is the most common and feared hospital-acquired electrolyte disorder in patients receiving intravenous fluid therapy.^1,2^ In the majority of hyponatremic patients, the iatrogenic decrease in *P*_Na_ is attributed to a disparity in electrolyte-free water intake and loss.^3^

There is a prevalent view that the administration of hypotonic fluid therapy is associated with the occurrence of hyponatremia in patients with high circulating plasma levels of antidiuretic hormone arginine-vasopressin (AVP).^4^ Consequently, the prescription of hypotonic fluid therapy to most hospitalized children is discouraged, particularly in the post-operative and medical acute care setting.^1^ As such, the choice and volume of optimal fluid therapy required for adequate fluid and electrolyte balance continues to challenge physicians.^5,6^

In a previous study, we observed that correction of extracellular fluid deficit with Ringer’s solution followed by hypotonic maintenance intravenous fluid therapy was not consistently associated with post-operative hyponatremia in children admitted with acute appendicitis.^7^ We also noted that in patients with high circulating plasma-AVP levels, the degree of post-operative water retention declined significantly, as patients became euvolemic.^7^ A limitation of this previous study was the lack of available data on urinary electrolyte and water balance required to determine clinically meaningful changes in *P*_Na_ concentration.

Considering that intravenous fluid administration is one of the most common therapies used in children in the perioperative period, we conducted this prospective observational single-center cohort study in previously healthy and normonatremic children admitted for suspected appendicitis and who received intravenous fluid therapy as per local guidelines. The primary objective of the current investigation was to assess changes in *P*_Na_ concentration directly after surgery. Additionally, we aimed to study key players in volume and osmoregulation as well as electrolyte and water balance and use these data to provide the physiological basis about the interpretation of the impact of intravenous fluid therapy on post-operative *P*_Na_.

## Methods

### Patients

The current study was conducted in Sweden, from 19 May 2021 until 28 February 2022. Ethical approval was given by The Swedish-Ethical Review-Authority (2020–03166). This study protocol was registered at the Australian New Zealand Clinical Trial Registry (https://www.anzctr.org.au ACTRN12621000587808). Before enrollment, all parents provided voluntary written informed consent for their children to participate in this study.

In our hospital, patients <10 years of age admitted for suspected appendicitis are transferred to another Hospital whereas patients ≥15 years of age are admitted to the adult medical ward. Therefore, the inclusion criteria were male or female previously healthy children ≥10 and <15 years of age that were admitted for suspected appendicitis and required urgent surgery. Exclusion criteria were hyponatremia, defined as a *P*_Na_ concentration <135 mmol/L, documented diagnosis of renal, endocrine, or metabolic disease, or having received intravenous fluid therapy before the admission at our hospital.

During the study period, 174 previously healthy children were admitted for suspected appendicitis. Still, the assessment of eligibility was limited to the shift when our dedicated study team was working, i.e., staff from the Department of Pediatric Emergency Medicine and from the Pediatric inpatient ward, Department of Surgery, and from the Department of Anesthesia and Intensive Care, respectively. With the onset of the pandemic, there was however a reorganization of hospital and medical spaces and staff to prioritize COVID-19–infected patients. This had a negative impact on the number of patients accrued in the study. Since the primary endpoint was to evaluate changes in *P*_Na_ from hospital admission to the end of surgery, we calculated the sample size based on our previously published data, in which *P*_Na_ values at the end of surgery where significantly lower as compared with admission plasma levels.^7^ We observed that the mean change between *P*_Na_ on admission and at the end of surgery was 3.5 mmol/L (*n* = 52; Std. Error 0.3, lower bound 2.7 and upper bound 4.3 mmol/L; *P*_Na_ on admission was 138.1 mmol/L (SD ± 2.7) and at the end of surgery was 134.6 mmol/L (SD ± 2.1), respectively). Based on this premise, we calculated the sample size for the current study using a power of 90% to detect a 3.5 mmol/L mean change in *P*_Na_ with an assumed standard deviation of 3.0 mmol/L, and with a two-sided controlled at the type-I error rate of 0.05. According to our calculation, a sample size of about 25 participants was needed to detect significant changes in *P*_Na_. Of the 23 recruited patients, 21 underwent surgery. However, none of them developed post-operative hyponatremia. In view of all these facts, the study was stopped earlier than planned.

### Fluid therapy

Since children managed for acute abdominal pain due to appendicitis usually have fever, nausea and/or vomiting, and poor fluid intake, we assumed that this group of patients are mildly dehydrated, i.e., <5% loss of body weight upon presentation at our Department of Pediatric Emergency Medicine.^8^ Therefore, and following our local recommendations for intravenous fluid therapy, all patients were prescribed a balanced crystalloid intravenous infusion at a dose of 50 mL/kg of Ringer’s acetate solution (131 mmol/L sodium, 4 mmol/L potassium, 2 mmol/L magnesium, 110 mmol/L chloride, 30 mmol/L acetate; Fresenius Kabi®) over 4 h. The infusion of this near-isotonic solution was followed by a maintenance fluid and electrolyte therapy phase consisting of a hypotonic 0.46% normal sodium chloride (80 mmol/L sodium, 20 mmol/L potassium, 100 mmol/L chloride in 5% glucose solution) until the start of the surgery. At the maintenance stage, infusion rate was decreased to 80% of normal maintenance fluid therapy. Normal maintenance fluid therapy was calculated according to the following empiric equations: for 0–10 kg = weight (kg) x 100 mL/kg/day, for 10–20 kg = 1000 mL + [weight (kg) x 50 mL/kg/day], and for >20 kg = 1500 mL + [weight (kg) x 20 mL/kg/day].^9^

All the patients were instructed to take nothing by mouth from admission until surgery.

During surgery, fluids were administered at the anesthetist’s discretion. All patients received intraoperative antibiotic prophylaxis. Anesthesia was induced with alfentanil, propofol, and suxamethonium and maintained with remifentanil and sevoflurane.

### Blood pressure measurement

All blood pressure measurements were performed by trained staff members by using an automatic oscillometric blood pressure device (GE Carescape V100 Dinamap Vital Signs Monitor) with the participants in supine position on admission, at induction of anesthesia, and directly after surgery, respectively. At each time point, three blood pressure measurements were taken, and its mean was used as the representative value.

### Electrolyte and water balance

Over the study period, i.e., from admission until the end of surgery, electrolytes (sodium, potassium, and chloride) and fluid intakes were calculated from intravenous fluid and medications, whereas electrolytes and fluid output were determined from collection of all urine. Before anesthesia, urine was obtained through spontaneous voiding and during anesthesia, urine was collected continuously by means of Foley urinary catheter, which was inserted after induction of anesthesia. Fluid lost from the respiratory system and skin (with or without fever), in the excreted stool, and from isolated vomiting episodes were not included. The electrolyte and water balances over the study period were calculated by subtracting total fluid output from total fluid input.

### Sample collections and laboratory analyses

On admission, before the start of fluid therapy, at induction of anesthesia, and immediately after surgery, *P*_Na_ together with plasma-potassium, chloride, creatinine, albumin, venous blood gas, blood hemoglobin, serum-osmolality, plasma-renin, plasma-aldosterone, plasma-AVP, plasma-nitrate, plasma-nitrite, and plasma-cyclic guanosine monophosphate (cGMP), were analyzed. Blood draw was performed in supine position. A spot urine sample was obtained on admission as well as directly after surgery and was analyzed for urine osmolality, creatinine, and sodium. In addition, all urine was carefully collected from admission until the end of surgery. The urine collection was recorded and measured volumetrically, and urine creatinine, sodium, potassium, and chloride concentration and urine osmolality were analyzed from an aliquot from the total collection.

Determination of routine laboratory tests were performed according to accredited hospital clinical laboratory procedure at the Department of Laboratory Medicine Södersjukhuset, Stockholm, Sweden. *P*_Na_ concentration was analyzed by indirect ion selective electrode.

Metabolic acidosis was defined by a pH less than 7.35 and a bicarbonate level below 22 mmol/L.

All samples for determination of plasma-renin, plasma-aldosterone, plasma-AVP, plasma-nitrate, plasma-nitrite, and plasma-cGMP were collected in EDTA tubes and processed to plasma (centrifugation at 3000 rpm, 10 min, 4 °C) and subsequently frozen at –80 °C until analysis. The analyses were performed blinded to participants’ characteristics.

### Plasma-nitrate and nitrite analysis

The plasma samples were extracted using HPLC grade methanol (CROMASOLV, Sigma-Aldrich). Subsequently, nitrite and nitrate levels were measured by a sensitive and selective measurement HPLC system (ENO-20 Eicom Japan), which uses reverse phase chromatography to separate nitrite from nitrate. Thereafter, nitrate was reduced to nitrite through a reaction with cadmium and reduced copper inside a reduction column. Reduced nitrite was then derivatized with Griess reagent and the level of diazo compounds were analyzed by detection at 540 nm as previously described in detail.^10^

### Plasma-cyclic guanosine monophosphate analysis

To prevent cGMP degradation, plasma for cGMP measurement was collected in an inhibitor of cAMP/cGMP phosphodiesterase; IBMX, 3-isobutyl-1-methylxanthine, 10uM (I7018; Sigma-Aldrich, Merck, Sweden). Plasma-cGMP concentration was analyzed, using a commercially available ELISA (Cayman Chemicals #581021 BioNordika, Sweden), according to manufactures instructions.

### Plasma-renin, aldosterone, and AVP analysis

As previously described,^7^ the plasma-renin concentration was determined from EDTA-plasma that was incubated with plasma from a nephrectomized sheep followed by radioimmunoassay of angiotensin I through the antibody trapping method as described by Poulsen and Jørgensen.^11^ Concentrations were measured by the rate of angiotensin I formation and standardized in terms of international units per liter (IU/L) based on the World Health Organization International Standard (ref. no. 68–356; National Institute for Biological Standards and Control, Hertfordshire, UK), with samples of 0.05 IU/L included in every run of the plasma-renin assay. The inter-assay coefficient of variation was 8%.

Plasma-aldosterone was determined by ELISA (MS E-5200, LDN, Labor Diagnostika Nord, Germany). Human EDTA-plasma pool was used as an internal inter-assay standard. The inter-assay coefficient of variation was 2.5%. Vasopressin levels were determined by radioimmunoassay as previously described,^12^ using a specific AVP antibody (AB3096).^13^

Peptides were extracted from plasma using Sep-Pak® Plus C18 extraction cartridges (Waters Corporation, Milford, MA). The detection limit was 0.10 pg/mL plasma, and the inter-assay coefficient of variation was 4.5%.

### Equations

The electrolyte-free water (mL) administered from admission until the end or surgery was calculated as follows: [Total volume infused (*L*) – (total sodium infused in mmol/*P*_Na_ at admission)] × 1000.

The fractional excretion of sodium (FE_Na_) was calculated from a spot urine sample taken on admission and at the end of surgery, as follows:

$${{{{{{{\mathrm{FE}}}}}}}}_{{{{{{{{\mathrm{Na}}}}}}}}} = \left[ {\left( {{{U}}_{{{{{{{{\mathrm{Na}}}}}}}}}/{{U}}_{{{{{{{{\mathrm{cr}}}}}}}}}} \right)/\left( {{{P}}_{{{{{{{{\mathrm{Na}}}}}}}}}/{{P}}_{{{{{{{{\mathrm{cr}}}}}}}}}} \right)} \right] \times 100$$

where *U*_Na_, *U*_cr_, and *P*_cr_ represents urinary sodium, creatinine, and plasma creatinine concentration, respectively.

Effective osmolal clearance (E_-_C_Osm_), which refers to the clearance of only effective osmoles, i.e., Na, K, and their accompanying anions was calculated as follows:^14^

$${{{{{{{\mathrm{E}}}}}}}}_ - {{{{{{{\mathrm{C}}}}}}}}_{{{{{{{{\mathrm{Osm}}}}}}}}}\left( {{{{{{{{\mathrm{mL}}}}}}}}/{{{{{{{\mathrm{min}}}}}}}}} \right) = {{U}}_{{{{{{{{\mathrm{Na}}}}}}}} + {{{{{{{\mathrm{K}}}}}}}}} \times {{V}}/{{P}}_{{{{{{{{\mathrm{Na}}}}}}}}}$$

where *U*_Na + K_ represents urinary sodium plus potassium concentration (mmol/L) from all urine that was carefully collected from admission until the end of surgery, and which was recorded and measured volumetrically; *V*, urinary flow rate (mL/min); *P*_Na_, plasma-sodium concentration at the end of surgery.

Electrolyte-free water clearance (${{{{{{{\mathrm{E}}}}}}}}_ - {{{{{{{\mathrm{C}}}}}}}}_{{{{{{{{\mathrm{H}}}}}}}}_2{{{{{{{\mathrm{O}}}}}}}}}$), a method used to determine whether net free water is reabsorbed from or added to the tubular fluid during urine concentration, and thereby its effect on *P*_Na_ was calculated as follows:

$${{{{{{{\mathrm{E}}}}}}}}_ - {{{{{{{\mathrm{C}}}}}}}}_{{{{{{{{\mathrm{H}}}}}}}}_2{{{{{{{\mathrm{O}}}}}}}}}\left( {{{{{{{{\mathrm{mL}}}}}}}}/{{{{{{{\mathrm{min}}}}}}}}} \right) = {{V}} - {{{{{{{\mathrm{E}}}}}}}}_ - {{{{{{{\mathrm{C}}}}}}}}_{{{{{{{{\mathrm{Osm}}}}}}}}}$$

### Statistical analysis

Statistical analyses were performed in R software, version 4.0.2. All continuous variables are presented as medians and interquartile ranges (IQR), unless otherwise stated. Paired *t*-test was carried out to compare individual differences between admission (baseline) and at the end of surgery, McNemar’s Chi-square test was used to assess the difference between two correlated proportions, and one-way repeated measure analysis of variance (ANOVA) followed by the post hoc Bonferroni correction, was performed for multiple pair-wise comparisons, i.e., between baseline, at induction of anesthesia, and at the end of surgery. Two-tailed *p*-values < 0.05 was considered statistically significant.

## Results

**Fig. 1:**
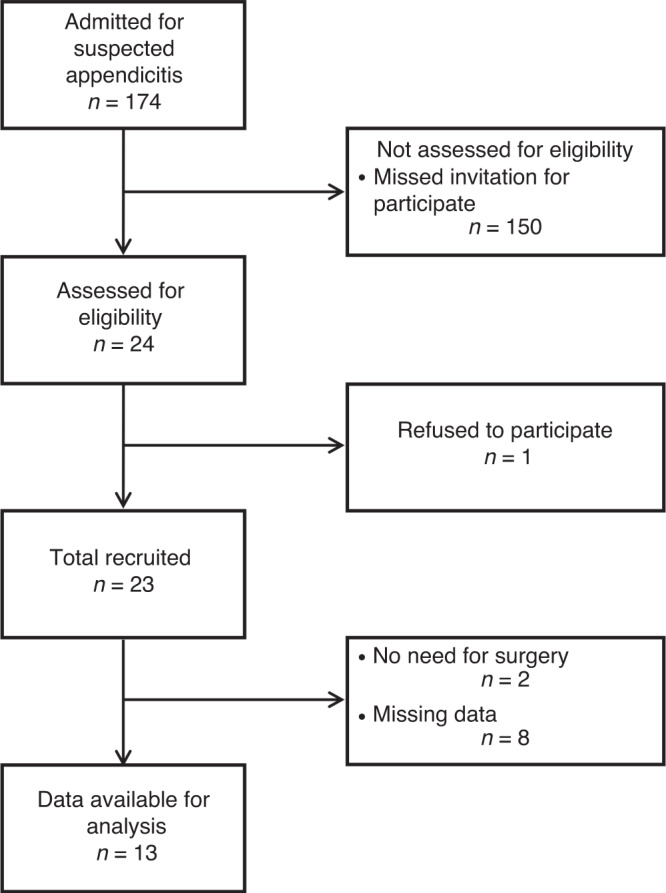
Flow chart of study population.

Out of the 23 patients enrolled in the study, 8 were excluded from the final analysis because of inadequate urine collection and/or measurement of urinary electrolyte losses during the study period, whereas 2 participants were excluded because surgical treatment was not indicated. Figure 1 shows the flow chart of the study participants. Our study population consisted therefore of 13 children (8 males) and had a median age of 12.3 (11.5–13.5) years. All the patients had post-operative histologically verified appendicitis: phlegmonous in 7 cases, gangrenous in 5 cases, and perforated in 1 case, respectively.^15^ The median time lapse from patients’ enrollment at hospital admission until the end of surgery was 985 (637–1130) minutes.

The median total fluid received from admission until the induction of anesthesia was 2280 (1900–3029) mL (40, 29–54 mL/kg). All participants received Ringer’s acetate solution; median 1900 (1000–2000) mL (35, 23–44 mL/kg). Four patients were operated after Ringer’s infusion whereas 9 patients received 0.46% normal sodium chloride as maintenance intravenous fluid therapy after Ringer’s acetate infusion while waiting for surgery (932, 672–1351 mL), respectively. Intraoperatively, 12 participants received Ringer’s acetate solution (295, 194–385 mL). The median volume of 0.9% saline solution (154 mmol/L sodium, 154 mmol/L chloride) used intraoperatively in all participants to administer medications, mainly antibiotics, was 267 (198–405) mL. Overall, the median fluid volume administered intraoperatively was 608 (394–812) mL (11, 10–14 mL/kg). The calculated median electrolyte-free water administered from admission until the end or surgery was 335 (92–446) mL. All patients with the exception of one participant received electrolyte-free water.

The median urine volume collected from admission until the end of surgery was 1200 (450–1850) mL (1.1, 1–1.9 mL/min). During the study period, the balance for water, sodium, and chloride was positive whereas it was negative for potassium (Table 1).

**Table 1:** Data on water and electrolyte balance (*n* = 13).

From admission to the end of surgery	Water (mL)	Sodium (mmol)	Potassium (mmol)	Chloride (mmol)
Infused	3117 (2478–3606)	358 (311–447)	21.9 (9–31.6)	355 (279–413)
Excreted	1200 (450–1850)	86.4 (48–146)	31.2 (8–54.6)	78 (48–136)
Balance	+1814 (1064–2120)	+270 (111–323)	–5.7 (–13.9 to –1.8)	+264 (137–285)

Results are presented as medians (IQRs).

The median E_-_C_Osm_ was 0.9 (0.7–1.6) mL/min and the median ${{{{{{{\mathrm{E}}}}}}}}_ - {{{{{{{\mathrm{C}}}}}}}}_{{{{{{{{\mathrm{H}}}}}}}}_2{{{{{{{\mathrm{O}}}}}}}}}$was 0.2 (0.1–0.2) mL/min, respectively. The ${{{{{{{\mathrm{E}}}}}}}}_ - {{{{{{{\mathrm{C}}}}}}}}_{{{{{{{{\mathrm{H}}}}}}}}_2{{{{{{{\mathrm{O}}}}}}}}}$was positive, indicating net water excretion, in all patients with the exception of two participants, in whom it was negative, –0.03 and –0.31 mL/min, respectively.

Table 2 shows the characteristics of the participants on admission, at induction of anesthesia, and at the end of surgery, respectively. There was a significant decrease in systolic and diastolic blood pressure, plasma albumin and in hemoglobin between time points (Table 2).

**Table 2:** Participants’ (*n* = 13) characteristics on admission, at induction of anesthesia, and at the end of surgery.

Variables	On admission	At induction of anesthesia	At the end of surgery
Weight, kg	50 (38–63)	NA	NA
Height, m	1.58 (1.53–1.68)	NA	NA
BMI, kg/m^2^	19.5 (17.4–22.1)	NA	NA
Systolic BP, mmHg	115 (108–120)	108 (103–113)	90 (85–94)^d^
Diastolic BP, mmHg	63 (58–66)	59 (57–64)	41 (39–46)^e^
Sodium, mmol/L	138 (137–139)	138 (137–138)	138 (137–139)
Potassium, mmol/L	4 (3.9–4.2)	4.2 (4–4.4)	4.5 (4.2–4.6)^f^
Chloride, mmol/L	102 (99–103)	104 (103–106)	105 (104–106)
Creatinine, µmol/L	46 (41–53)	49 (40.8–54.5)	54 (41–60)^g^
Albumin, g/L^a^	39 (37–40)	34 (31–42)^i^	31 (29–32)^h^
Hemoglobin, g/dL	130 (122–132)	114 (111–123)^k^	109 (103–116)^j^
Bicarbonate, mmol/L^b^	25 (24–25)	24 (22–24)	22 (21–23)^l^
S-Osm, mOsm/kg	287 (285–288)	287 (284–289)	289 (287–290)
U-Osm, mOsm/kg	637 (531–933)	NA	416 (332–525)^m^
FE_Na_, %^c^	0.3 (0.2–0.4)	NA	0.7 (0.4–0.7)^n^

Results are presented as medians (IQRs) unless otherwise indicated. *NA* not available, *BMI* body mass index, *BP* blood pressure, *S-Osm* serum-osmolality, *U-Osm* urine osmolality, *FE*_*Na*_ fractional excretion of sodium.

^a^Albumin data were available in 10 and 12 patients on admission and at induction, respectively.

^b^Bicarbonate data were available in 12, 10, and 11 patients on admission, at induction, and at the end of surgery, respectively.

^c^FE_Na_ data were available in 10 of the 13 patients.

Post hoc analysis (Bonferroni test):

^d^*p* < 0.001 vs. admission and *p* < 0.01 vs. induction, respectively;

^e^*p* < 0.0001 vs. admission and vs. induction;

^f^*p* < 0.05 vs. admission;

^g^*p* < 0.05 vs. induction;

^h^*p* < 0.001 vs. admission;

^i^*p* < 0.01 vs. admission;

^j^*p* < 0.0001 vs. admission and *p* < 0.01 vs. induction, respectively;

^k^*p* < 0.01 vs. admission;

^l^*p* < 0.05 vs. admission. Paired *t*-test:

^m^*p* = 0.2;

^n^*p* = 0.052.

At the end of surgery, plasma-potassium values were significantly higher as compared with admission plasma levels, and venous blood bicarbonate levels were significantly lower as compared with admission values. In addition, plasma creatinine levels at the end of surgery were found to be significantly higher as compared with plasma values at induction of anesthesia (Table 2).

Metabolic acidosis, on admission, at induction of anesthesia, and at the end of surgery, was observed in none, one, and three participants, respectively. Low FE_Na_ values (<0.5%), which indicate sodium retention by the kidneys as a mechanism to preserve the extracellular volume,^16,17^ were found in 8 patients on admission, and in 4 patients at the end of surgery, respectively (*p* = 0.13).

Overall, there were no significant decreases in *P*_Na_ levels over the study period and none of the patients became hyponatremic at the end of surgery.

In 11 of the 13 patients, determinations of plasma-renin, aldosterone, AVP, nitrite, nitrate, and cGMP were performed on admission, at induction of anesthesia, and at the end of surgery, respectively (Table 3). At the end of surgery, plasma-renin, aldosterone, and nitrite levels were significantly higher as compared with admission plasma levels, and plasma-renin levels at induction of anesthesia were significantly higher as compared with admission values (Table 3). Plasma-AVP levels at induction of anesthesia were significantly lower as compared with admission values (Table 3). Plasma-AVP values > 4 pg/mL, which are expected to produce maximum antidiuresis,^18^ were observed in 64%, 45%, and 45% of the participants on admission, at induction of anesthesia, and at the end of surgery, respectively.

**Table 3:** Plasma-renin, aldosterone, arginine-vasopressin, nitrite, nitrate, and cyclic guanosine monophosphate levels over the study period in 11 participants.

Variables	On admission	At induction of anesthesia	At the end of surgery
Renin (mIU/L)	40 (19–59)	177 (60–239)^a^	261 (175–301)^b^
Aldosterone (pg/mL)	97 (36–118)	136 (91–262)	173 (136–422)^c^
AVP (pg/mL)	5.1 (3.5–18.6)	3.6 (1.9–7.8)^d^	3.8 (1.9–7.8)
Nitrite (µmol/L)	0.07 (0.04–0.1)	0.15 (0.11–0.37)	0.96 (0.72–1.01)^e^
Nitrate (µmol/L)	12.7 (10.8–15.3)	10.7 (9.7–13.2)	11.9 (11–13)
cGMP (pmol/mL)	18.9 (13.5–28.1)	17 (13.8–28.1)	21.1 (17.1–23.4)

Results are presented as medians (IQRs).

*AVP* arginine-vasopressin, *cGMP* cyclic guanosine monophosphate.

Post hoc analysis (Bonferroni test):

^a^*p* < 0.05 vs. admission and

^b^*p* < 0.001 vs. admission, respectively;

^c^*p* < 0.05 vs. admission;

^d^*p* < 0.05 vs. admission;

^e^*p* < 0.05 vs. admission.

## Discussion

In the present study, we observed that the use of moderately hypotonic fluid therapy after correction of extracellular fluid deficit is not necessarily associated with post-operative hyponatremia. All participants except one received electrolyte-free water. The latter, in the presence of high circulating plasma-AVP levels on admission, might have had a clinically meaningful impact on *P*_Na_ levels at the end of surgery.^18^ Of note, almost all of the patients were able to excrete net free water, which is essential in preventing clinically important changes in *P*_Na_ concentrations.^19^ This is also reflected in the assessment of water and electrolyte balance (Table 1), which shows that the amount of water and sodium retained was rather isotonic, thus preventing the appearance of hyponatremia.

Of equal importance to mention is that when the patients were admitted to the Emergency Department, all were assumed to be mildly dehydrated. Our study design precluded us from asserting the precise percentage of extracellular fluid deficit at admission, since we did not have the differences between ill weights and well weights, which is the most reliable clinical method to assess the percentage of fluid loss.^20,21^ However, even with this limitation, medical history of poor fluid intake before admission, low FE_Na_ values and increased urine osmolality on admission, positive water balance in addition to a marked dilution of plasma albumin and hemoglobin levels observed over the study period, sodium retention as well as a negative balance for potassium, accompanied by increased circulating levels of aldosterone together with increased plasma-AVP levels on admission all suggest that a state of dehydration was present when participants entered hospital.^22^ In parallel to the aforementioned, we observed a significant decrease in blood bicarbonate levels at the end of surgery (Table 2). This observation, in the presence of no significant changes in plasma chloride,^23^ indicates a diluted bicarbonate due to volume expansion.^24^

If our premise is accepted as true, then our data support previous published studies, in which a physiologically based argument for fluid therapy was proposed to lessen the risk of hospital-induced hyponatremia.^25–28^ The authors advocate that in children admitted to hospital because of acute illness the administration of intravenous maintenance fluids such as hypotonic saline should not be started unless the extracellular fluid deficit has first been restored with rapidly infused isotonic saline or Ringer’s solution.^25–28^ Thus, we speculate that in our patients net free water excretion should have occurred after their extracellular fluid deficit had been corrected. In our previous investigation as well as in the current study,^7^ fluid deficit was corrected with a balanced crystalloid solution. In patients with normal renal function, the prescription of intravenous fluid therapy given at full maintenance rate may be potentially unsafe when renal water excretion is limited by AVP excess. Therefore, intravenous maintenance fluid therapy after Ringer’s acetate infusion was prescribed at a lower rate than the recommended average maintenance rate in both studies.^28^ In the present study, in contrast to our previous investigation, we used 0.46% instead of 0.23% normal sodium chloride in 5% glucose as maintenance fluid therapy. In the latter investigation, we observed that 17 out of 44 participants that were normonatremic on admission became hyponatremic at the end of surgery. Therefore, we cannot exclude that the use of 0.46% normal sodium chloride might have accounted for the lack of post-operative hyponatremia episodes seen in this study. This line of reasoning is supported by data from recently published randomized clinical trials conducted in acutely ill children,^29^ including patients before appendectomy.^30^ In these trials, participants were allowed to receive intravenous bolus of fluid replacement as either isotonic crystalloid or as a balanced crystalloid solution if clinically indicated. It was showed that the risk of hyponatremia was not increased in patients who received intravenous maintenance fluid therapy as 0.45% saline compared with patients that received 0.9% saline or 0.46% saline as compared with patients that received 0.81% saline fluid therapy, respectively.^29,30^ Whether the use of the foregoing-moderately hypotonic fluid therapy after correction of fluid deficit should be regarded as a *“transition phase treatment”* rather than a *“maintenance phase”* deserves further attention. The significant decrease in circulating plasma-AVP levels at induction of anesthesia as compared with admission levels noted in the current study provides the basis of this line of thinking.

We also observed that blood pressure levels were significantly lower at the end of surgery as compared with blood pressure measurements taken on admission and at induction of anesthesia (Table 2). As discussed above, it is unlikely that our patients were hypovolemic before the induction of anesthesia. In addition, all participants received intravenous fluid intraoperatively, and the amount of blood lost during the surgical procedures was negligible (data not shown). In this regard, the observed post-operative decrease in blood pressure was most likely due to other factor(s) rather than to patients’ fluid status. Indeed, the hemodynamic response to anesthetics, including the involvement of endogenous vasoactive substances such as vasodilator nitric oxide and reduced sympathetic nerve activity are a well-described phenomenon.^31–33^ We observed that plasma levels of nitrite, an oxidized circulating metabolite of nitric oxide, increased significantly after surgery, which might in fact indicate a rise in nitric oxide formation.^32^ This observation is in line with the aforementioned anesthetics-mediated effects on nitric oxide formation and metabolism.^31,32^ Even more importantly, studies conducted in animals have shown that nitric oxide modulates water and electrolyte handling along nephron partly via non-cGMP-dependent mechanisms,^34^ and decreases AVP-stimulated water and sodium reabsorption in the collecting duct.^35^ It is also noteworthy to mention that in rats exposed to a continuous intravascular infusion of l-deamino-8-arginine-vasopressin,^36^ which may increase nitric oxide formation,^37^ plasma volume expansion was accompanied by a progressive increase in urine flow and decreases in urine osmolality and free water reabsorption despite the presence of AVP,^36^ a phenomenon known as renal “escape”.^38,39^ Taken together, these data suggest that the process of escape from AVP-induced antidiuresis might have occurred in our patients.^40^ Additional investigations will be however required to assess this possibility.

Furthermore, a number of hormonal changes occur in response to surgery, which may influence electrolyte and water metabolism.^41^ Our results are in line with the aforementioned studies, since we also observed that the plasma-renin and aldosterone levels were significantly higher at the end of surgery as compared with their preoperative levels (Table 3). In a recently published randomized clinical study we also noticed that patients anesthetized with sevoflurane showed increased post-operative plasma-renin and plasma creatinine levels as compared to propofol anesthesia.^42^ According to previous investigations, the pronounced impairment in renal excretory function by sevoflurane anesthesia seem most likely to be due to increased renal sympathetic nerve activity, which under experimental conditions remains high even after fluid resuscitation.^43^ It is also recognized that the increase in renal sympathetic activity and release of norepinephrine is responsible for an increase in renal afferent arteriolar constriction and activation of beta-adrenergic receptor on juxtaglomerular cells,^44,45^ leading to an increase in renin secretion and, ultimately, an increase in aldosterone secretion.^46^ Even though it cannot be proven in this study, we reason that the use of sevoflurane anesthesia might have resulted in a decrease in renal blood flow and glomerular filtration rate and therefore accounted for the significant increase in plasma creatinine levels and renin observed in our patients at the end of surgery.

Although no patient was identified as having developed hyperkalemia, defined as plasma-potassium levels >5.5 mmol/L, we observed that post-operative plasma-potassium levels were significantly higher as compared to plasma levels obtained on admission. The occurrence of post-operative elevated plasma-potassium levels is however a well-described phenomenon, and its pathogenesis is multifactorial.^47^

It is worth mentioning that previously published clinical data have shown that sustained post-operative plasma elevations of AVP may result in clinically meaningful water retention after surgery.^48^ The authors demonstrated, in presumably euvolemic children that underwent elective surgery, that the risk of hyponatremia was significantly greater in patients who received 0.45% saline as compared with 0.9% saline solution. The events of hyponatremia occurred in the first 24 h after surgery in the majority of affected participants and plasma-AVP levels were elevated in both treatment arms on post-operative day 1.^48^ Similarly, in another controlled trial,^49^ the administration of 0.45% saline as compared with 0.9% saline solution in children after abdominal surgery was a risk factor for hyponatremia. These results are undeniably relevant. However, they should not be compared with ours since the data is derived from normohydrated patients who did not require urgent surgery and where the intervention was conducted after surgery.

The current study has inherent limitations. Firstly, the sample size is small, due to a combined consequence of missing data and poor participant recruitment. Secondly, this is a single-center study design. Therefore, additional investigations are needed to confirm our observations. However, it was encouraging to find that our results may provide a functional explanation of *P*_Na_ levels over time that have been reported in larger and more inclusive randomized controlled intervention studies that also used moderately hypotonic intravenous fluid therapy in acutely ill children.^29,30^

Finally, in order to avoid hospital-acquired hyponatremia, intravenous fluids therapy should be closely monitored and regarded as a potent medicine that is administered either to restore circulation, to correct a fluid deficit, or to preserve the normal body water volume and its electrolyte composition.

## Data Availability

The datasets generated during the current study are available from the corresponding author on reasonable request.
