# Correction: Perioperative water and electrolyte balance and water homeostasis regulation in children with acute surgery

**DOI:** 10.1038/s41390-023-02975-7

**Published:** 2023-12-19

**Authors:** Daniel N. Roberts, Paula Vallén, Maria Cronhjort, Tobias Alfvén, Gabriel Sandblom, Susanna Törnroth-Horsefield, Boye L. Jensen, Per-Arne Lönnqvist, Robert Frithiof, Mattias Carlström, Rafael T. Krmar

**Affiliations:** 1 Sachsska Children and Youth Hospital, Stockholm, Sweden; 2 Department of Anesthesia and Intensive Care, Södersjukhuset, Stockholm, Sweden; 3 Department of Clinical Science and Education, Södersjukhuset, Karolinska Institutet, Stockholm, Sweden; 4 Department of Global Public Health, Karolinska Institutet, Stockholm, Sweden; 5 Department of Surgery, Södersjukhuset, Stockholm, Sweden; 6 Department of Biochemistry and Structural Biology, Lund University, Lund, Sweden; 7 Department of Cardiovascular and Renal Research, Institute of Molecular Medicine, University of Southern Denmark, Odense, Denmark; 8 Department of Urology, Odense University Hospital, Odense, Denmark; 9 Department of Pediatric Perioperative Medicine and Intensive Care, Astrid Lindgren Children’s Hospital, Karolinska University Hospital, Stockholm, Sweden; 10 Department of Physiology and Pharmacology, Karolinska Institutet, Biomedicum 5B, Stockholm, Sweden; 11 Department of Surgical Sciences, Anesthesiology, and Intensive Care, Uppsala University, Uppsala, Sweden

Correction to: *Pediatric Research* 10.1038/s41390-023-02509-1, published online 09 February 2023

In the original article, the author name Susanna Törnroth-Horsefield was incorrectly given as ‘Susanna Tönroth-Horsefield’. The original article has been corrected.

